# Building alliances for early detection of immunodeficiencies: from primary care to hematology

**DOI:** 10.3389/fimmu.2025.1701384

**Published:** 2026-01-02

**Authors:** Jacques G. Rivière, Marlène Pasquet, Eleonora Gambineri

**Affiliations:** 1 Universitat Autònoma de Barcelona (UAB), Barcelona, Spain; 2 Infection and Immunity in Pediatric Patients Research Group, Vall d’Hebron Institut de Recerca (VHIR), Barcelona, Spain; 3 Pediatric Infectious Diseases and Immunodeficiencies Unit, Hospital Infantil i de La Dona Vall d’Hebron, Vall d’Hebron Barcelona Hospital Campus, Barcelona, Spain; 4 Jeffrey Modell Diagnostic and Research Center for Primary Immunodeficiencies, Barcelona, Spain; 5 Pediatric Haematology and Immunology, Toulouse University Hospital, Toulouse, France; 6 University of Toulouse, Toulouse Cancer Research Center (CRCT), INSERM U1037, IUCT-Oncopole, Toulouse, France; 7 Department of Neuroscience, Psychology, Drug Research, and Child Health (NEUROFARBA) Department, University of Florence, Florence, Italy; 8 Department of Haematology/Oncology, Meyer Children’s Hospital, IRCCS, Florence, Italy

**Keywords:** artificial intelligence, autoimmune cytopenias, early diagnosis, inborn errors of immunity, lymphoproliferative disorders, PIDCAP project, primary care

## Abstract

Inborn errors of immunity (IEI), also known as primary immunodeficiencies, are a heterogeneous group of rare disorders characterized by increased susceptibility to infections, immune dysregulation, and malignancy. Early detection remains a major challenge due to the complexity of clinical presentations, limited awareness among non-specialists, and delayed diagnostic pathways. This review explores current strategies to enhance early detection of IEI, highlighting both technological innovations and clinical insights. Tools such as newborn screening, the Jeffrey Modell Foundation (JMF) warning signs, software like SPIRIT, and the PIDCAP project—a structured model designed for primary care implementation using ICD-coded clinical data— have shown promise in identifying at-risk patients. Artificial intelligence (AI) offers additional potential by detecting diagnostic patterns in electronic health records, although challenges related to data quality, heterogeneity, and system interoperability persist. Importantly, hematologic manifestations such as autoimmune cytopenias, lymphoproliferative disorders, and myelodysplastic syndromes often precede or accompany IEI and should prompt immunological evaluation. These conditions, frequently encountered in hematology, may serve as early clinical clues and justify genetic and immunophenotypic assessment. A multidisciplinary approach combining primary care, immunology, hematology, and AI technologies is essential to advance the early detection of IEI. Projects like PIDCAP, and their potential extension to secondary immunodeficiencies, exemplify scalable, patient-centered strategies that may significantly improve diagnostic timeliness and clinical outcomes.

## 1 Introduction

Inborn errors of immunity (IEI), also known as primary immunodeficiencies (PIDs), were traditionally considered rare diseases, affecting approximately 1/10,000 – 1/50,000 births (1–3). However, recent data suggest that, collectively, IEIs are more common than previously thought. A retrospective study in the United States estimated a prevalence of 6/10,000 individuals (4), while global estimates suggest that at least 1% of the population may be affected by IEI, although prevalence varies significantly across countries (5). These conditions primarily manifest through severe and recurrent infections, but thanks to an increased access of next-generation sequencing, a broader spectrum of phenotypes associated with IEI has been identified. These include immune dysregulation, syndromic features, and malignancy, which together account for approximately 30% of initial clinical manifestations, underscoring the need for broader clinical suspicion beyond infection alone (6).

The heterogeneity in clinical phenotypes means that patients often need to consult multiple medical specialties, starting with general practitioners and including hematologists, among others. Beyond hematologic signs, other non-infectious manifestations —such as allergy, organ-specific autoimmunity, granulomatous-lymphocytic interstitial lung disease, granulomatous dermatitis, arthritis, and inflammatory bowel disease— are increasingly recognized as part of the IEI spectrum (7–9). This complexity is compounded by limited knowledge of IEI among non-immunologists and insufficient resources, which are key factors contributing to delays in diagnosis (10–12). Consequently, up to 50% of patients present with some degree of organ damage at the time of diagnosis (13, 14), highlighting the need for timely intervention to prevent further complications.

This review focuses on the tools available for the detection of IEI and the clinical warning signs that can pave the road to build alliances with general practitioners and hematologists to achieve early detection of IEI.

## 2 Screening immunodeficiencies at the frontline

### 2.1 Tools for screening IEI

There are various tools and initiatives developed to improve the early detection of IEI. Newborn screening has proven highly effective, allowing for prompt intervention (15–17). The Jeffrey Modell Foundation (JMF) warning signs have been a valuable resource for over 20 years, aiding in the identification of potential IEI (18). Additionally, the SPIRIT (Software for Primary Immunodeficiency Recognition, Intervention, and Tracking) analyzer, a software tool developed by the JMF and others, was designed to recognize, intervene, and track IEI (19). The PIDCAP project (Primary Immune Deficiency Centre d’Atenció Primària [primary care center]) focuses on enhancing early detection efforts in primary care centers (20). The use of Human Phenotype Ontology (HPO) enhances genetic diagnosis by providing a comparable standardized vocabulary of phenotypic abnormalities (21). Furthermore, artificial intelligence (AI) models are being explored to gather and analyze more data, potentially improving screening outcomes (22–25). These tools collectively aim to facilitate early detection and intervention, thereby preventing the progression of organ damage in patients with IEI.

### 2.2 Innovations and challenges in AI for IEI

AI is often heralded as a revolutionary tool, sometimes referred to as the fourth industrial revolution (26). However, at the user level in its application into healthcare, its effectiveness can be limited. For instance, when presented with a patient summary and tasked with providing a diagnosis, AI’s performance is notably less reliable, particularly for rare diseases such as IEI (27, 28).

**Figure 1:**
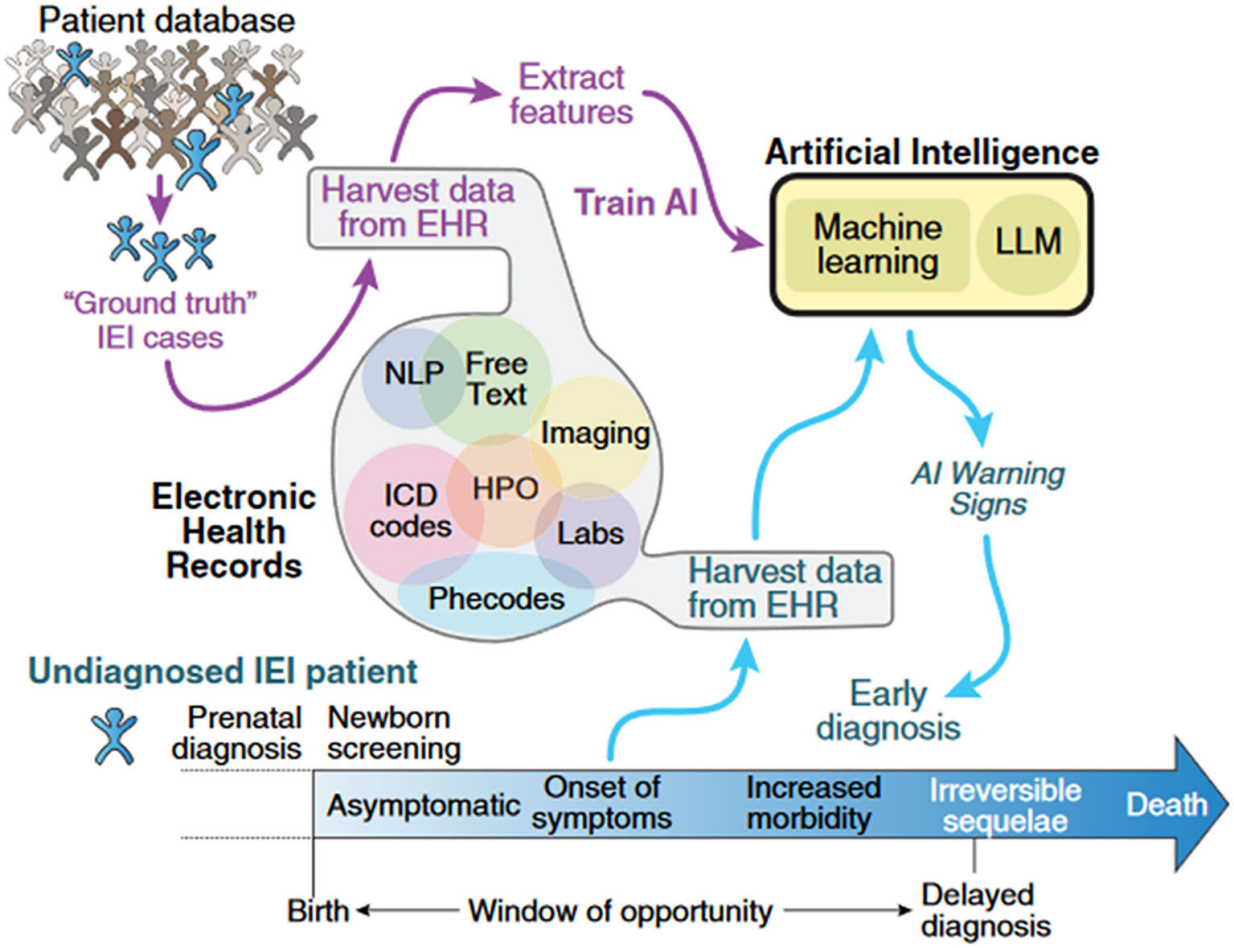
How artificial intelligence can help in the early diagnosis of IEI. This conceptual diagram illustrates the potential of AI to identify undiagnosed patients with IEI by leveraging structured and unstructured data from EHR. AI models —including machine learning, LLMs, and NLP— can extract features from clinical notes, laboratory results, ICD codes, HPO terms, and phecodes to detect diagnostic patterns. By training on confirmed IEI cases (“ground truth”), these systems can flag high-risk individuals before symptom onset or during early disease stages, enabling timely referral and intervention. The figure highlights the critical “window of opportunity” between birth and the onset of irreversible complications, emphasizing the role of AI in preventing delayed diagnosis and improving outcomes. AI, artificial intelligence; EHR, Electronic Health Record; HPO, Human Phenotype Ontology; ICD, International Classification of Diseases; IEI, inborn errors of immunity; LLM, Large Language Model; NLP, Natural Language Processing. From Rivière et al. (29): Rivière JG, Soler Palacín P, Butte MJ. J Allergy Clin Immunol. 2024;153 (3):637-642.

The journey from a patient’s first contact with the healthcare system to the initial suspicion of an IEI diagnosis often involves multiple missed opportunities, as these conditions present numerous symptoms and frequent interactions with healthcare providers before a diagnosis is made. By analyzing medical records, AI can detect patterns that suggest IEI. This approach can lead to earlier diagnosis and treatment, which improves patient outcomes and reduces costs. Current methods typically involve training AI algorithms on the clinical features of known IEI cases, which can then be applied to new patients to identify potential diagnoses more quickly and accurately (**Figure 1**) (29).

Over the past two years, AI has experienced notable advancements, particularly in the transition from unimodal to multimodal models (30). Traditionally, AI systems operated on a unimodal basis, where the input and output were of the same type, such as text-to-text or video-to-video. However, recent developments have focused on integrating diverse data types, including text, video, and sensor data, to produce more comprehensive outputs. These multimodal models represent a significant innovation in AI, potentially leading to more effective and nuanced solutions, enhancing the capability for early detection and improved management of complex conditions, such as IEI.

Despite these advancements in AI, its application in healthcare, particularly in IEI, faces several challenges (29, 31, 32). One major issue is the quality and structure of data, which is often scattered and unstructured, complicating AI analysis. The lack of standardization and varying data models across different vendors further exacerbates this problem, making it difficult to integrate and utilize data effectively. Additionally, cultural barriers such as insufficient training for healthcare professionals and the prevalent practice of copy-pasting data reduce the overall quality of electronic health records (EHR) (33, 34). Organizational challenges also play a significant role, as the lack of time for proper documentation hinders the accurate and efficient use of data for AI applications. These factors could collectively stop the potential of AI to revolutionize healthcare (33).

### 2.3 Approaches to improving IEI detection

The rapid growth and interest in the fields of AI have driven significant advancements in healthcare. Notably, extensive research is being conducted on the application of AI in IEI. Recent key contributions have validated the use of EHR data to estimate IEI prevalence and risk, developed AI-based risk prediction tools for patients with recurrent infections, and proposed a validated AI-based process for IEI screening (4, 31, 35). Other significant works include the development of an algorithm for early detection of patients at risk of primary antibody deficiency in a primary care setting, exploration of AI for IEI, the use of natural language processing for early risk ascertainment, and the identification of undiagnosed patients with common variable immunodeficiency (CVID) disease through EHR signatures (22, 24, 36, 37).

In general, there are two complementary and non-exclusive approaches to improving IEI detection, the expert-based approach, and the data-based approach. The expert-based approach benefits from high expertise, easier access to unpublished data, and lower implementation cost. However, it is harder to reproduce, has a narrower data reach, and is slower to implement. On the other hand, the data-based approach, while more limited in expertise, might offer broader data reach, quicker implementation, and easier reproducibility and learning capacity. Nevertheless, it faces challenges such as harder access to unpublished data, higher susceptibility to data misinterpretation, and greater difficulty in overcoming EHR data limitations. The primary aim of both data-driven and expert-driven methods is to develop models that serve as practical and accessible tools, aiding clinical decision-making and potentially enhancing the early diagnosis and treatment of patients.

AI has generated considerable interest as a potential tool for improving IEI detection. While retrospective studies and proof-of-concept models have shown promising results, AI application in real-world clinical workflows remains limited (38). Therefore, it should be considered an emerging tool under validation rather than an established screening method. To date, no sustained prospective initiatives have demonstrated AI-driven alerts leading directly to confirmed IEI diagnoses, highlighting the need for further research before widespread implementation (39).

Recent retrospective studies have validated the ability of AI to correctly identify previously diagnosed cases of IEI. Machine learning models integrating clinical variables with traditional criteria have significantly improved early detection of IEI. For example, tools using PheNet have showed potential to anticipate CVID diagnoses by over a year (22), and population-wide pipelines have shown similar performance (31). Real-world applications are also emerging, including AI-assisted flow cytometry, risk prediction systems like PI Prob, and personalized treatment strategies based on multi-omics integration (35, 40, 41). Despite these advances, challenges remain regarding data quality, phenotypic heterogeneity, and implementation, which must be addressed to ensure broader clinical applicability (25, 42).

### 2.4 PIDCAP project

Primary care physicians are ideally positioned to initiate diagnostic suspicion of IEI. Their awareness of these rare conditions is crucial to reducing diagnostic delays (43). However, due to the vast array of general diseases and the complexity and rarity of IEI, it is unrealistic to expect primary care physicians to maintain comprehensive awareness (20, 44). Recognizing this, the PIDCAP project has developed a scoring system for use in primary care settings (20, 45).

The PIDCAP project is an initiative aimed at identifying individuals at high risk of IEI using ICD (International Classification of Diseases) codes within the Catalan healthcare system. An expert-based approach was employed in this project, utilizing warning signs and updating them to create a simple yet effective model. This model is practical and can be integrated into advanced AI systems or used as a standalone clipboard method (20).

A key feature of the PIDCAP project is the involvement of primary care physicians from the outset. These physicians are intimately familiar with the data they input into the EHR, including quality and limitations of this data, and the patients they treat regularly. Their involvement ensures that any tool developed is both practical and implementable in real-world scenarios (20).

The project started by reviewing the 10 warning signs for IEI published by the JMF (46) and expanded by harvesting additional warning signs from the literature (47–49). Local experts in Catalonia and Spain were consulted to reach a consensus on these warning signs. Each warning sign was then mapped to corresponding ICD codes, resulting in the identification of approximately 3,000 codes for pediatric warning signs and a similar number for adults (20).

A Delphi-like survey was conducted to validate these warning signs, with participating medical experts asked to evaluate each one. If a warning sign was deemed important (a score of 75 points of more), it triggered an immediate alert to the primary care healthcare professional and suggested a referral to an immunologist (20).

**Figure 2:**
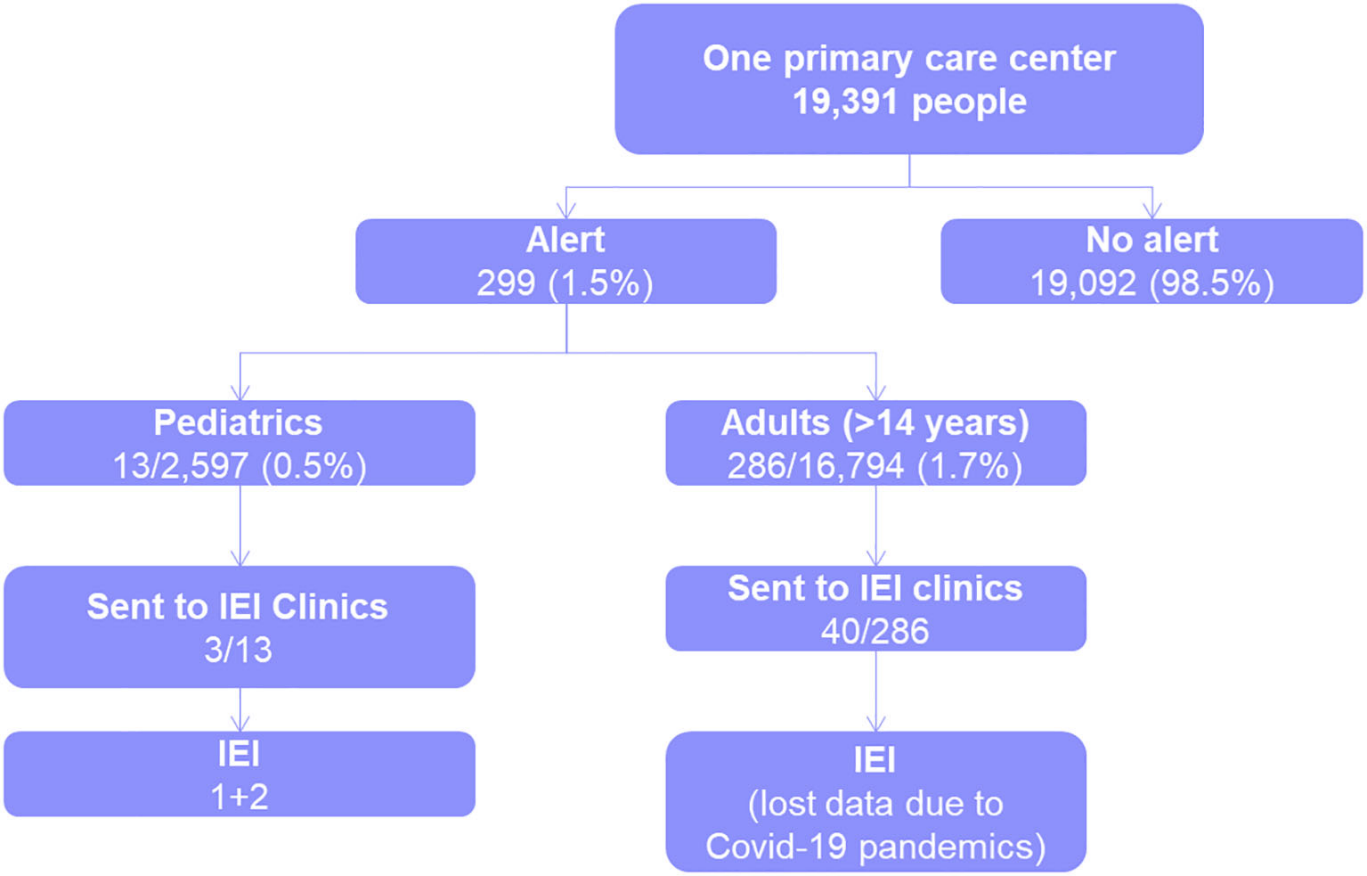
Real-world testing of the PIDCAP scoring system. Pilot implementation of the PIDCAP scoring system in a primary care center serving 19,391 people. The system generated alerts for 1.5% of the population (299 patients), with notably low alert rates in pediatrics (0.5%) and adults (1.7%), minimizing false positives and alert fatigue. Among alerted patients, a subset was referred to immunodeficiency clinics and confirmed IEI diagnoses were made in several cases. These findings demonstrate the feasibility and specificity of PIDCAP in routine clinical workflows. Data loss due to the COVID-19 pandemic affected follow-up in some cases. IEI, Inborn Errors of Immunity.

The PIDCAP system was implemented in a pilot area covering about 20,000 residents in Barcelona (16,794 adults and 2,597 children). The system generates alerts within the EHR when a patient meets the criteria for IEI screening. These alerts provide detailed information on the warning signs and the patient’s history, including the triggering ICD-10 codes, the overall risk score, and recent laboratory data such as blood counts and immunoglobulin levels. Physicians are presented with three actionable options: to request a basic immunological workup, to initiate a virtual or in-person referral to a specialist, or to dismiss the alert if deemed not clinically relevant. Despite the challenges posed by the COVID-19 pandemic, which halted primary care activities for two years, the project demonstrated a low alert rate (0.5% in pediatrics and 1.7% in adults), ensuring that physicians were not overwhelmed by false positives (**Figure 2**). The workflow was well received by primary care professionals, who reported that the system was useful and non-intrusive. As a result, 40 adult and 3 pediatric patients were referred for further immunological evaluation before stopping due to the pandemic. The top discriminating warning signs included not only well-known indicators like recurrent bronchiectasis and infections but also newer signs such as autoimmune diseases and chronic diarrhea. All warning signs included in the final pediatric and adult scoring system are shown in **Table 1** (20). These early results support the feasibility and acceptability of the PIDCAP model in real-world settings and provide a strong foundation for its broader implementation and prospective evaluation.

**Table 1:** Warning signs included in the final pediatric and adult scoring system.

Warning signs for pediatric patients	Warning signs for adult patients
≥ 10 acute otitis media	≥ 8 acute otitis media
≥ 3 sinusitis or orbital cellulitis	≥ 8 sinusitis or chronic sinusitis
≥ 3 pneumonia	≥ 3 pneumonia
Failure to thrive	Chronic diarrhea
Deep abscesses (in organs)	Deep abscesses (in organs and/or ganglia)
≥ 3 recurrent skin abscesses	Recurrent skin abscesses of repetition (≥ 3)
Mucocutaneous candidiasis (oropharynx, cutaneous, excluded vaginal) in patients ≥ 12 months of age: ≥ 2 episodes	Oropharyngeal or cutaneous candidiasis (excluding vaginal candidiasis)
≥ 2 systemic infections (including sepsis)	Recurrent viral infections (colds, herpes, warts, condylomas, etc.) ≥ 25 episodes
≥ 1 serious infection that alone indicate IEI study (meningitis caused by HSV, etc.)	≥ 2 systemic infections including sepsis
Family history of inborn errors of immunity	Unique severe condition that alone require study for inborn errors of immunity
Consanguinity or other family history compatible with manifestations of inborn errors of immunity (lymphomas, etc.)	Atypical mycobacteria infection
Cytopenia (not specified as autoimmune)	Family history of inborn errors of immunity
Autoimmune cytopenia	Presence of ≥ 2 warning signs
Presence of ≥ 2 warning signs	Consanguinity or other family history compatible with manifestations of inborn errors of immunity (haematological neoplasms)
Systemic autoimmune diseases, not including autoimmune cytopenia (celiac disease, arthritis, etc.)	Presence of cytopenia (without specifying if autoimmune)
Endocrinopathology: Hypothyroidism, hyperparathyroidism, diabetes, etc. (Not described as autoimmune)	Autoimmune cytopenia
Hematological malignancy	Presence of bronchiectasis without cystic fibrosis
Solid organ neoplasia (only those that have been associated with inborn errors of immunity in pediatrics: thyroid)	Systemic and endocrine autoimmune diseases (celiac disease, arthritis, systemic lupus, thyroiditis, etc.)
Oral (dental/palatal) anomalies	Haematological neoplasia (excluding multiple myeloma, chronic myeloid leukemia, Waldenström's disease, etc)
Chronic diarrhea; or ≥ 10 episodes of acute diarrhea	Solid organ neoplasia (only those related with inborn errors of immunity: skin, stomach, thyroid)
Chronic viral skin infection; or ≥ 20 acute episodes	Inflammatory bowel disease
Chronic eczema or other dermatological manifestations related to inborn errors of immunity	Recurrent fever
Recurrent fever	Oral (dental/palatal) anomalies
Inflammatory bowel disease in patients ≥ 2 years of age	Chronic eczema or other dermatological manifestations related to inborn errors of immunity
Inflammatory bowel disease in patients < 2 years of age	
Bronchiectasis without cystic fibrosis	
Vaccine reaction	

(Adapted from Rivière et al. (20))

### 2.5 Global expansion and future directions of the PIDCAP project

The PIDCAP project is poised for global expansion. This expansion involves collaboration with scientific societies such as the Clinical Immunology Society (CIS), the European Society for Immunodeficiencies (ESID), and the International Nursing Group for Immunodeficiencies (INGID) leveraging their expertise to refine and validate the warning signs for IEI through a Delphi questionnaire. Moreover, the involvement of patient advocacy groups, such as the International Patient Organization for Primary Immunodeficiencies (IPOPI), further underscores the project’s commitment to patient-centered care. This diverse group includes not only physicians but also nurses and expert patients, ensuring a comprehensive perspective on IEI. This phase of the project is conducted under the umbrella of the JMF, a pioneer in the field of warning signs in IEI. The project’s future directions include the development of a federated model to facilitate data sharing and comparison across different regions while ensuring data protection. This model aims to enable the use of PIDCAP’s tools in various locations, such as Barcelona, the United States, and other parts of Europe, thereby assessing the project’s scalability and effectiveness on a global scale.

Additionally, the PIDCAP project is exploring the application of its methodologies to secondary immunodeficiencies (SID). Historically, SID has been primarily associated with adults undergoing treatments like immunosuppressive therapies for hematological malignancies, autoimmune conditions, or transplantation, while IEI were considered genetic conditions. However, recent insights reveal a significant overlap between primary and secondary immunodeficiencies (50). The project aims to leverage the IEI knowledge for screening SID, although the behavior of available screening tools in the context of SID remains uncertain. Furthermore, SID faces complexities introduced by the rapid evolution of treatments and the combination of drugs (40, 51–58). Therefore, the objective is to leverage the tools developed for IEI to apply them to SID. By utilizing the large datasets available for SID, particularly through AI, we can gain insights that may also benefit IEI. The project also emphasizes the importance of systematic screening of patients before initiating immunosuppressive treatments and conducting prompt genetic studies if SID is diagnosed, drawing parallels with practices in IEI management (48, 49).

## 3 Clues to immunodeficiencies in the hematology scenario

In hematology and oncology, there is a growing recognition of the link between immunodeficiency and cancer. Hematological conditions, such as autoimmune cytopenias and lymphoproliferative disorders (LPD), may serve as indicators of IEI. While there are scattered reports in the literature, including case reports by Ballow M et al. and Chandra S et al. (50, 59), comprehensive multicenter studies are still lacking.

**Figure 3:**
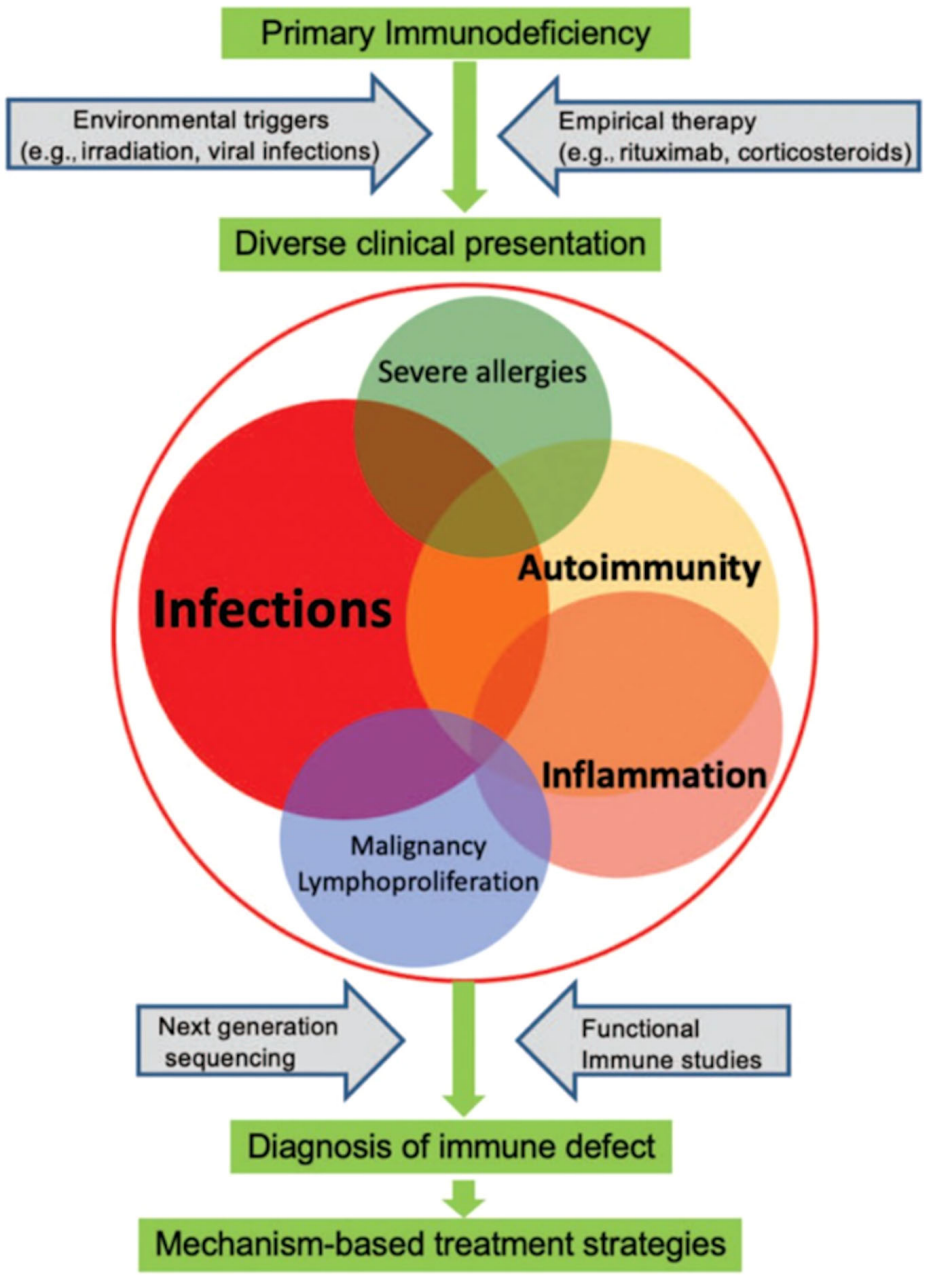
Clinical presentations of IEI. The broad and heterogeneous clinical spectrum of inborn errors of immunity (IEI) may include infections, autoimmunity, inflammation, lymphoproliferation, malignancy, and severe allergies. Environmental triggers and empirical therapies often precede diagnosis, which is typically achieved through next-generation sequencing and functional immune studies. Early recognition of these diverse manifestations is essential to guide mechanism-based treatment strategies. From Walte et al. (60): Walter JE et al. Curr Opin Pediatr 2019;31 (6):851-862.

Awareness of the diverse clinical manifestations of IEI is essential, as timely diagnosis and treatment are critical for improving patient outcomes. IEI are no longer defined solely by recurrent or severe infections; instead, non-infectious complications such as autoimmunity, chronic inflammation, lymphoproliferation, and malignancy are increasingly recognized as part of their clinical spectrum (**Figure 3**) (6, 60). This expanding phenotype brings IEI into closer focus within the hematology and oncology landscape.

### 3.1 Cytopenias

**Figure 4:**
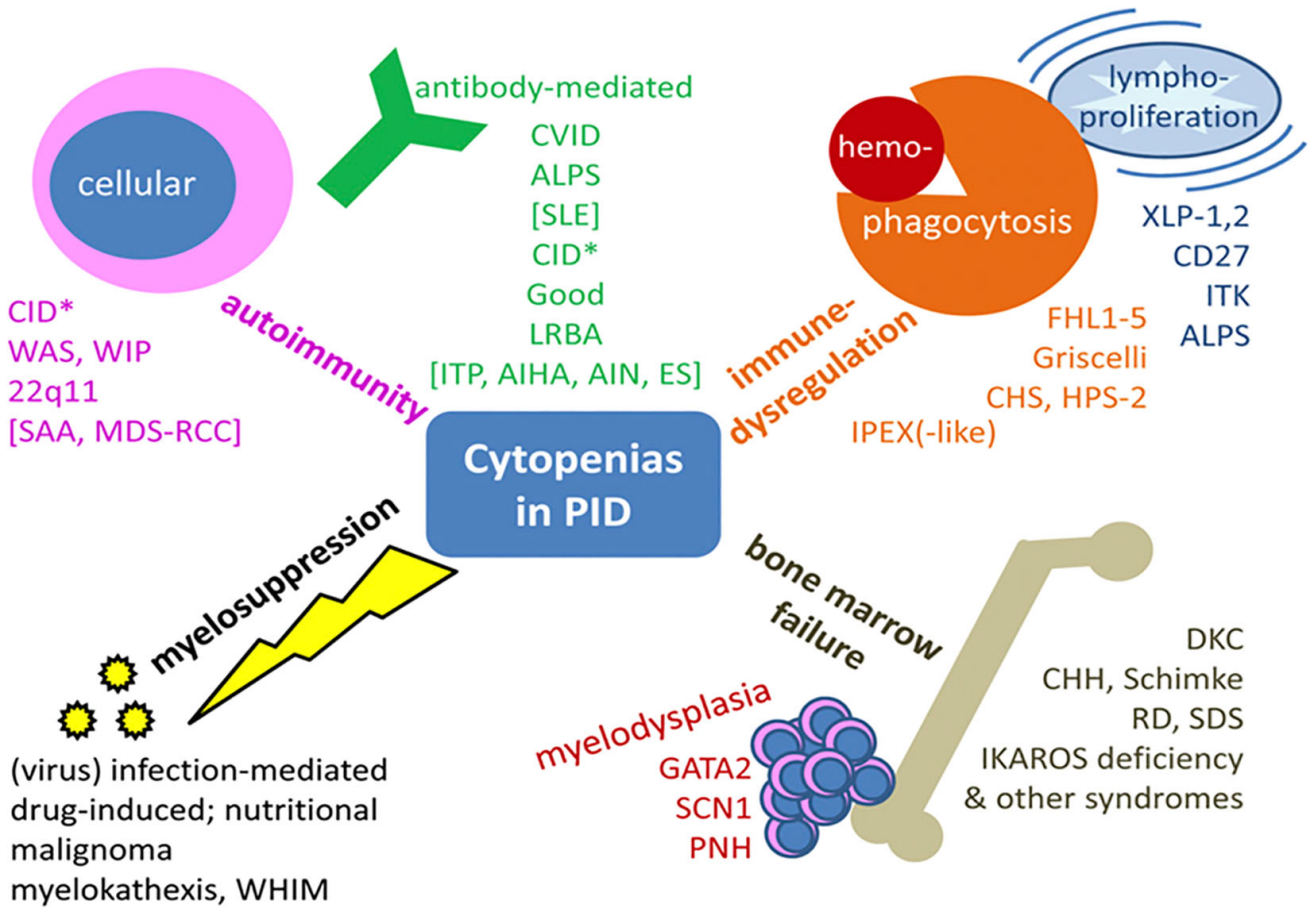
Mechanisms of cytopenias in IEI. This figure summarizes the diverse and overlapping mechanisms leading to cytopenias in IEI, including autoimmunity (e.g., ITP, AIHA, AIN), immune dysregulation (e.g., ALPS, LRBA, IPEX-like syndromes), lymphoproliferation, bone marrow failure syndromes (e.g., GATA2, DKC, SDS), and myelosuppression due to infections, drugs, or nutritional deficiencies. These mechanisms reflect the complexity of IEI-related hematologic manifestations and highlight the need for integrated immunological and genetic evaluation. AIHA, autoimmune hemolytic anemia; AIN, autoimmune neutropenia; CHH, cartilage hair hypoplasia; CHS, Chediak-Higashi syndrome; DKC, dyskeratosis congenita; FHL1-5, familial hemophagocytic lymphohistiocytosis 1-5; HPS-2, Hermansky-Pudlak syndrome 2; IEI, inborn errors of immunity; ITK, IL-2–inducible T-cell kinase deficiency; LRBA, lipopolysaccharide-responsive beige-like anchor deficiency; PNH, paroxysmal nocturnal hemoglobinuria; RCC, refractory cytopenia of childhood; RD, reticular dysgenesis; SCN1, severe congenital neutropenia 1; SDS, Shwachman-Diamond syndrome; WHIM, warts, hypogammaglobulinemia, immunodeficiency, myelokathexis; WIP, WAS protein-interacting protein; XLP-1,2, X-linked lymphoproliferative disease 1,2. From Seidel (61): Seidel MT. Blood 2014;124 (15):2337-44.

IEI-related cytopenias can arise through various mechanisms, including antibody-mediated autoimmunity, immune dysregulation (such as hyperinflammation and lymphoproliferation), bone marrow failure (BMF), and myelosuppression (**Figure 4**) (61). These mechanisms often overlap, complicating the clinical distinction between IEI, SID, hematologic malignancies, and autoimmune diseases.

**Figure 5:**
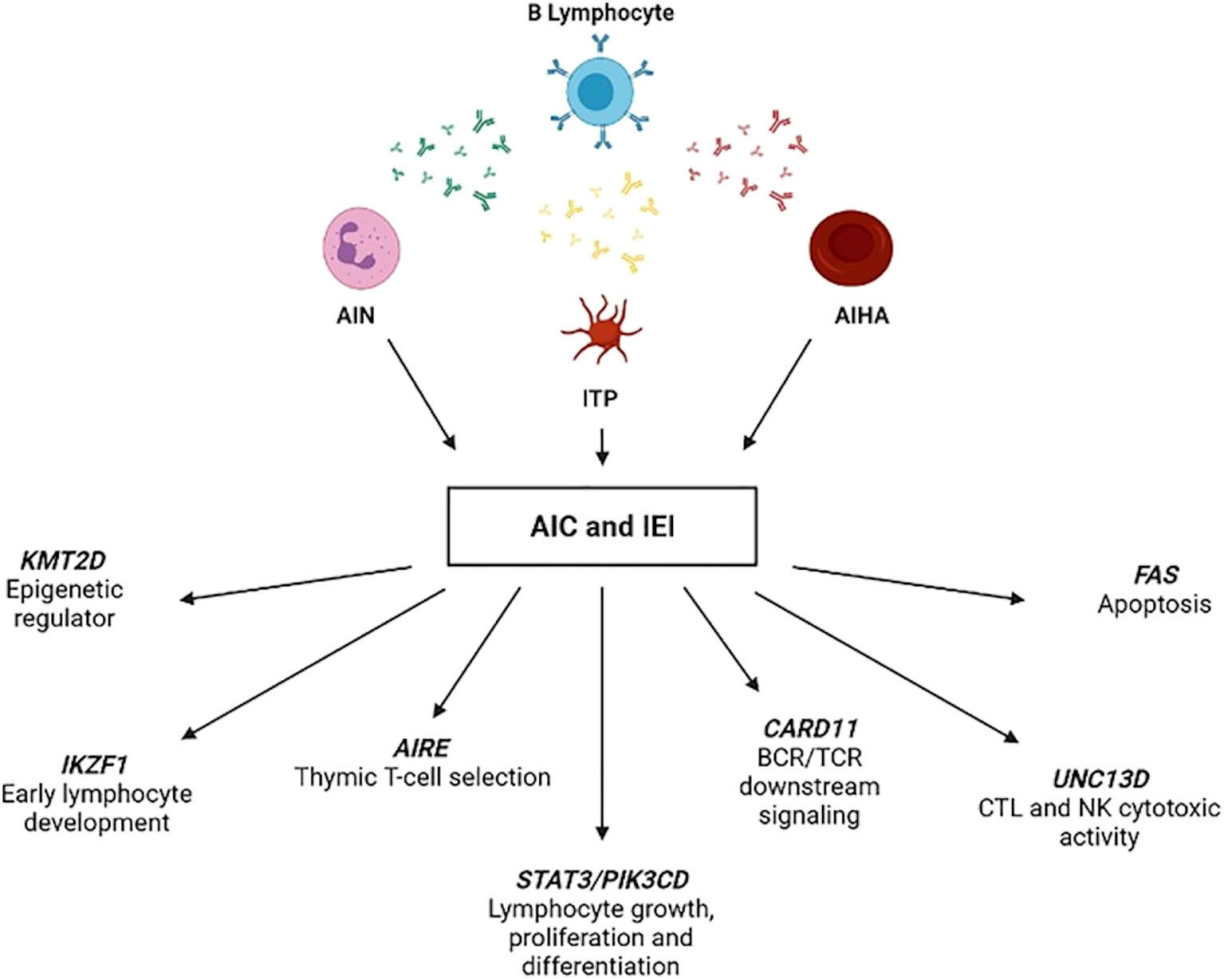
IEI-associated gene variants identified in autoimmune cytopenias patients with signs of IEI. This figure illustrates representative gene variants found in patients with AIC who also exhibited immunological features suggestive of IEI. The genes shown are involved in diverse immunological pathways, including apoptosis (FAS), thymic selection (AIRE), lymphocyte development (IKZF1), cytotoxic function (UNC13D), and signaling cascades (CARD11, STAT3, PIK3CD). These findings highlight the genetic heterogeneity underlying AIC and reinforce the importance of immunogenetic evaluation in patients with atypical or refractory cytopenias. AIC, autoimmune cytopenia; AIN, autoimmune neutropenia; AIHA, autoimmune hemolytic anemia; IEI, inborn errors of immunity; ITP, immune thrombocytopenia. From Schiavo E et al. (68): Schiavo E et al. Front.Immunol 2022:12:790455.

Autoimmune cytopenias are recognized as the main clinical presentation of autoimmunity within the field of IEI (62). Chronic and treatment-refractory forms are particularly suggestive of an underlying immune defect (61, 63–67). Recent studies emphasize the value of comprehensive immunophenotyping, including the evaluation of immunoglobulin levels (e.g., IgA deficiency or elevated IgM), and genetic testing in this patient population. For instance, Schiavo et al. conducted a prospective study involving 30 patients with various autoimmune cytopenias associated, some of whom exhibited signs of IEI. The study revealed significant imbalances in the naïve and memory T-cell compartments among autoimmune cytopenias patients and signs of IEI, characterized by lower naïve T-cells and higher effective central memory T-cells. Pathogenic or likely pathogenic variants in IEI-related genes were identified in 53% of patients, affecting diverse immunologic pathways. This highlights that cytopenias can have diverse mechanisms of action related to IEI (**Figure 5**). Importantly, these patients also showed improved outcomes following immunomodulatory therapy, further reinforcing the clinical relevance of identifying underlying genetic defects (68). This and similar studies (69) underscore the need for a comprehensive immunological assessment in patients with atypical or refractory cytopenias to identify potential genetic mutations associated with IEI. The identification of such mutations can guide targeted therapies, thereby improving patient outcomes.

From a clinical standpoint, hematologists should consider an underlying IEI in patients presenting with early-onset, chronic, relapsing, or treatment-refractory cytopenias, particularly when associated with immune dysregulation, lymphoproliferation, or a personal or family history of recurrent infections (70, 71). Basic immunological screening—including complete blood count, immunoglobulin levels, and lymphocyte subsets—should be performed at first presentation, ideally before initiating immunosuppressive therapies such as rituximab, which may alter immunophenotypic profiles (71, 72).

### 3.2 Hematological malignancy as a red flag for immune deficiency

#### 3.2.1 Lymphoproliferative disorders

Lymphadenopathy is a common finding in several IEI, particularly those involving immune dysregulation or autoinflammatory conditions. Moreover, polyclonal lymphoproliferation, which manifests as lymphadenopathy and hepatosplenomegaly, may result from dysregulated immune or inflammatory responses. In fact, lymphadenopathy can represent the first clinical presentation or the leading sign of immune disorders. This is typical in syndromes such as autoimmune lymphoproliferative syndrome (ALPS), activated PI3K delta syndrome (APDS), and Epstein-Barr virus (EBV)-associated conditions (73).

In a cohort of approximately 50 children with non-malignant lymphoproliferation, Forbes et al. found that 60% harbored pathogenic or likely pathogenic variants in genes related to immune regulation, lymphocyte activation, or apoptosis, including *FAS, CTLA4, and LRBA* (74). Furthermore, patients with genetically confirmed IEI had better 10-year survival estimates, highlighting the prognostic and therapeutic implications of early genetic diagnosis, particularly with regard to curative interventions such as hematopoietic stem cell transplantation (74).

Despite these insights, distinguishing between LPDs, lymphoma, and IEI remains challenging, particularly when considering transitions from polyclonal to clonal proliferation. Currently, no standardized monitoring guidelines exist for patients with LPD and suspected IEI, even though they carry an elevated risk of lymphomagenesis. The integrity of germinal centers and the architecture of lymphoid follicles are critical in determining the immune system’s “fitness” and may inform risk assessment. While specific biomarkers for lymphoproliferation are generally limited, some exceptions exist ─ such as elevated double-negative T cells in ALPS and reduced switched memory B cells or elevated serum BAFF levels in CVID. These examples illustrate how certain IEIs can provide valuable insights into lymphoproliferative activity. Diagnostic imaging and biopsies are essential tools in this process. Moreover, targeted treatments and precise diagnoses are vital for improving patient outcomes, and the diagnosis of IEI will significantly impact the treatment strategy and prognosis.

**Figure 6:**
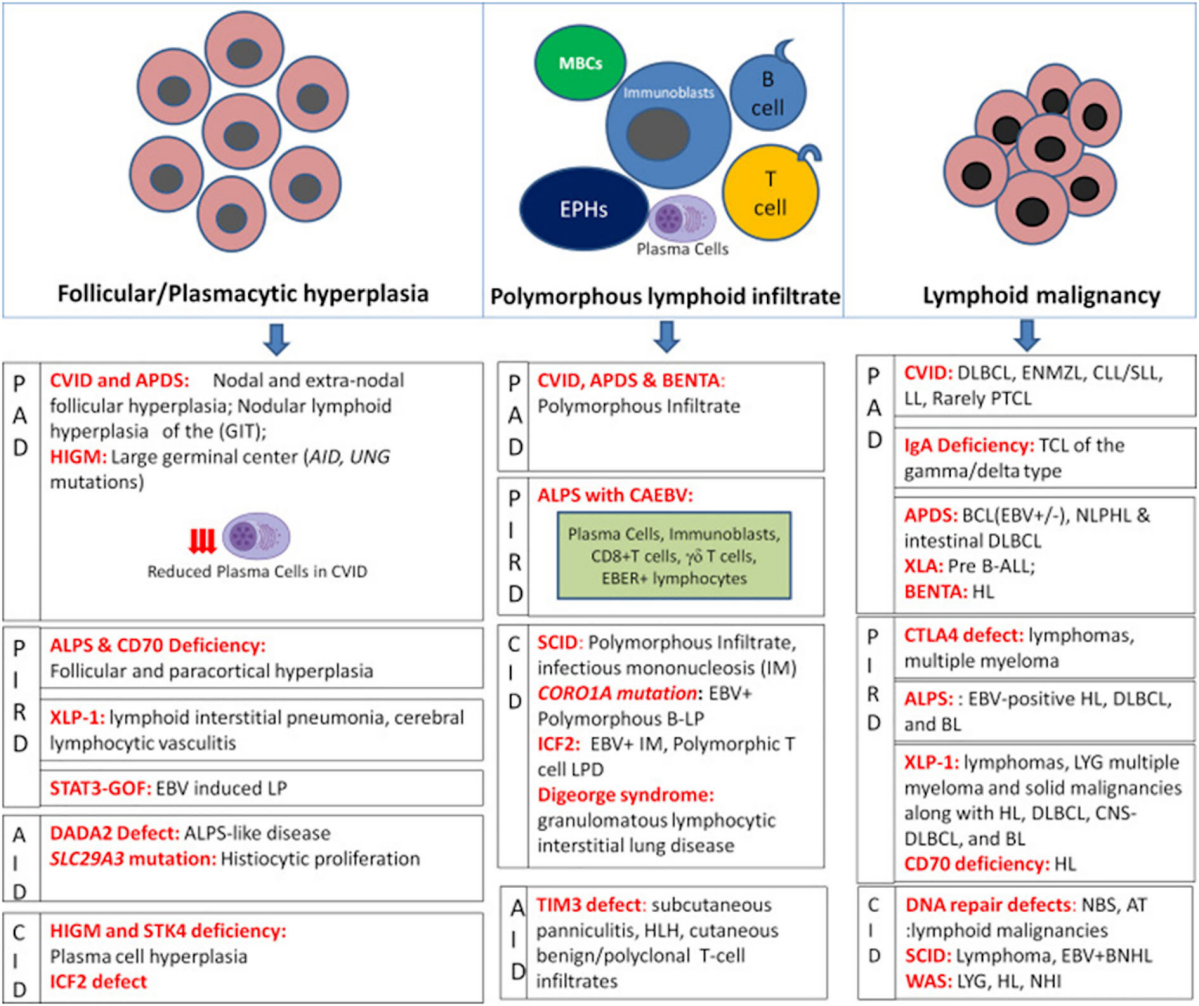
Histopathological alterations in lymphoproliferation associated with IEI. This figure illustrates the diverse histopathological patterns observed in lymphoid tissues of patients with IEI. These include follicular and plasmacytic hyperplasia, polymorphous lymphoid infiltrates, and nodal or extranodal lymphoid malignancies. Specific IEIs such as CVID, APDS, ALPS, and XLP-1 are associated with distinct features like reduced plasma cells, EBV-positive infiltrates, and atypical germinal center architecture. The figure also highlights the overlap between immune dysregulation and lymphomagenesis, with examples of DLBCL, HL, and other malignancies arising in the context of IEI. These histological findings underscore the importance of integrating immunological and genetic data in the evaluation of lymphoproliferative disorders. AID, autoimmune deficiency; *AID*, activation-induced cytidine deaminase; ALPS, autoimmune lymphoproliferative syndrome; APDS, activated phosphoinositide 3-kinase delta syndrome; AT, ataxia-telangiectasia; B-ALL, B-cell acute lymphoblastic leukemia; BCL, B-cell lymphoma; BENTA, B cell expansion with NF-κB and T cell anergy; BL, Burkitt lymphoma; BNHL, B-cell non-Hodgkin's lymphoma; CAEBV, chronic active EBV infection; CID, combined immunodeficiency; CLL/SLL, chronic lymphocytic leukemia/small lymphocytic lymphoma; CNS, central nervous system; CTLA-4, cytotoxic T-lymphocyte–associated antigen 4; CVID, common variable immunodeficiency; DADA2, Deficiency of adenosine deaminase 2; DLBCL, diffuse large B-cell lymphoma; EBER, EBV-encoded RNA; EBV, Epstein-Barr virus; ENMZL, extranodal marginal zone lymphoma; EPH, epithelioid histiocyte; HIGM, hyper IgM syndrome; HL, Hodgkin lymphoma; HLH, hemophagocytic lymphohistiocytosis; ICF2, Immunodeficiency with centromeric instability and facial anomalies type 2; IEI, inborn errors of immunity; IM, infectious mononucleosis; LL, lymphocytic leukemia; LP, lymphoproliferation; LPD, lymphoproliferative disorder; LYG, lymphomatoid granulomatosis; MBC, monocytoid B-cell; NBS, Nijmegen breakage syndrome; NHL, non-Hodgkin lymphomas; NLPHL, nodular lymphocyte-predominant Hodgkin lymphoma; PAD, primary antibody deficiency; PIRD, primary immune regulatory disorders; PTCL, peripheral T-cell lymphoma; SCID, severe combined immunodeficiency; STAT3-GOF, signal transducer and activator of transcription 3 gain-of-function; STK4, Serine/threonine-protein kinase 4; TCL, T-cell lymphoma; TIM3, T-cell immunoglobulin and mucin domain-containing protein 3; *UNG*, uracil DNA glycosylase; WAS, Wiskott-Aldrich syndrome; XLA, X-linked agammaglobulinemia; XLP, X-linked lymphoproliferation. From Sharma S et al. (75): Sharma S et al. Front Immunol 2022;13:856601.

Sharma et al. aimed to classify various immune defects by analyzing the histopathology of lymph nodes. However, significant overlaps exist, as conditions like CVID and APDS can both present with follicular hyperplasia ─ an expansion of germinal centers due to chronic immune stimulation ─ or polymorphous infiltrates, which refer to a heterogeneous mix of immune cells infiltrating the lymph node (**Figure 6**) (75). This histological overlap complicates the ability to definitively associate specific patterns with particular diseases. Nevertheless, future research may yield molecular signatures that enhance diagnostic specificity and deepen our understanding of lymphoproliferative presentations in IEI.

In patients with non-malignant lymphoproliferation, especially when associated with autoimmune features or cytopenias, an underlying IEI should be considered. A detailed medical history, including recurrent or severe infections, is essential to guide suspicion (70).

#### 3.2.2 Lymphomas

In the context of IEI, lymphomas are particularly noteworthy due to their association with immune dysregulation. A study by Xiaofei et al. analyzed tumor and germline DNA from 23 patients with suspected immunodeficiency and lymphoma. This study identified disease-causing germline mutations in 14 patients, primarily associated with CVID and APDS (*ATM, BACH2, BLM, CD70, G6PD, NBN, PIK3CD, PTEN, TNFRSF13B*). Additionally, somatic mutations were profiled, revealing eight genes with significantly higher mutation rates in IEI-associated diffuse large B-cell lymphomas (DLBCLs) compared to non-IEI DLBCLs (*BRCA2, NCOR1, KLF2, FAS, CCND3, and BRWD3*) (76).

Recent findings by Palacios-Ortega et al. further underscore the diagnostic complexity of lymphomas in the context of IEI. In a cohort of 151 patients with SID and B-cell lymphoproliferative disorders, including non-Hodgkin lymphoma, a significant proportion (70%) were reclassified as having suspected late-onset IEI based on clinical and immunological criteria. Notably, these patients exhibited higher rates of childhood infections, lower serum free light chain levels, and reduced switched memory B cells, features commonly associated with CVID. Genetic screening revealed that over 66% of the suspected IEI group carried at least one variant related to IEI, with many involving genes implicated in immune dysregulation and DNA repair (77).

**Figure 7:**
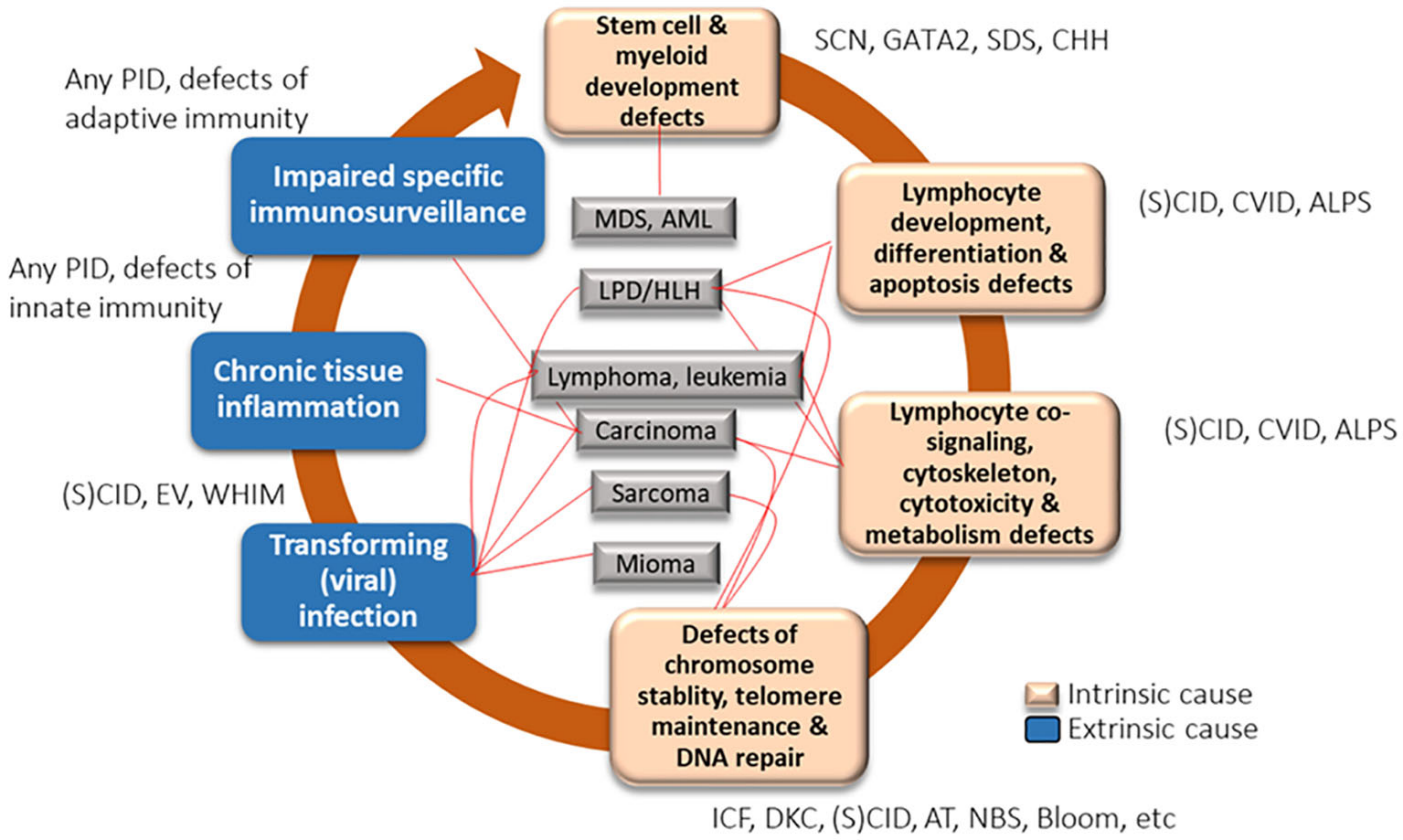
Factors implicated in the development of hematological malignancies in IEI. This figure outlines the intrinsic and extrinsic mechanisms contributing to hematological malignancies in patients with IEI. Intrinsic factors include defects in stem cell and myeloid development (e.g., GATA2, SDS), lymphocyte differentiation and apoptosis (e.g., CVID, SCID), and genomic instability (e.g., ICF, DKC, AT). Extrinsic contributors encompass chronic inflammation, impaired immunosurveillance, and transforming viral infections (e.g., EBV, HPV), which can lead to lymphomas, leukemias, and other solid tumors. The interplay between immune dysregulation and defective DNA repair pathways underscores the elevated cancer risk in IEI. ALPS, autoimmune lymphoproliferative syndrome; AML, acute myelogenous leukemia; CVID, common variable immune deficiency; DKC, dyskeratosis congenita; EV, epidermodysplasia verruciformis; IBD, inflammatory bowel disease; ICF, immunodeficiency with centromeric instability and facial anomalies; IEI, inborn errors of immunity; LPD, lymphoproliferative disorder; MDS, myelodysplastic syndrome; PID, primary immunodeficiency; (S)CID, (severe) combined immunodeficiency; SDS, Shwachman-Diamond syndrome; SM, smooth muscle tumor; WHIM, warts, hypogammaglobulinemia, immunodeficiency, and myelokathexis. From Ballow et al. (50): Ballow M et al. Front Immunol. 2022:13:928062.

These findings support the notion that pathogenesis of lymphoma in IEI involves both intrinsic factors —such as predisposing germline mutations— and extrinsic contributors, including viral infections, chronic inflammation, and cumulative somatic mutations (**Figure 7**). Recognizing this dual influence is vital for developing effective surveillance and personalized treatment strategies for these high-risk patients (50).

Identifying a concomitant IEI in patients with lymphoma is clinically relevant, as it may influence therapeutic decisions, including the choice between autologous and allogeneic hematopoietic stem cell transplantation. Allogeneic hematopoietic stem cell transplantation is generally preferred due to its potential to correct the underlying immune defect and provide a curative approach, especially in severe cases of IEI. However, the decision must be personalized, considering the specific IEI diagnosis, genetic mutations, and the patient’s overall clinical condition, with a multidisciplinary approach being essential for optimal outcomes (76, 78–84).

#### 3.2.3 Myelodysplastic disorders/bone marrow failure

Myelodysplastic syndrome (MDS), while predominantly an adult disease, also presents in children with an annual incidence of 1.8–4 cases per million (85). Pediatric MDS commonly presents with thrombocytopenia and neutropenia and is often secondary to inherited predisposition syndromes such as Fanconi anemia, dyskeratosis congenita, and Shwachman-Diamond syndrome (85). Notably, GATA2 and SAMD9/SAMD9L syndromes are the most common predisposing conditions in pediatric MDS (86).

The progression to MDS/acute myelogenous leukemia is driven by an accumulation of somatic mutations, often on a background of germline defects. Current research aims to identify specific germline mutations that accelerate this process using induced pluripotent stem cells technology and gene-editing tools (87). These inherited mutations increase the likelihood of acquiring somatic changes within hematopoietic stem cells, which can be primed to inflammation (so called ‘inflammaging’), linking BMF and immune dysregulation. Therefore, understanding the germline mutations that predispose individuals to MDS can provide insights into disease mechanisms and potential therapeutic targets (86).

A strong association exists between autoimmune disorders and MDS/BMF with a variety of interface disorders traditionally equated with MDS or IEI when in fact they are disorders due to errors in the function of both the bone marrow and the immune system. Hence, the traditional separation of cytopenias into mutually exclusive categories of “destruction” versus “production” is no longer consistent. Instead, a multidisciplinary approach involving pathologists, hematologists, and immunologists is essential for accurate diagnosis and management (88).

## 4 Conclusions

The timely diagnosis of IEI is crucial for improving patient treatment and outcomes and preventing severe complications. This review highlights the significant advancements and tools available for early diagnosis, including newborn screening, the JMF warning signs, and software like SPIRIT. The integration of AI in analyzing medical records shows promise, although challenges such as data quality and standardization remain for its implementation at larger scale. It is important to note that AI remains as an experimental tool rather than an established screening method, and prospective validation is needed before AI can be considered a reliable screening tool.

The PIDCAP project exemplifies a successful initiative in primary care settings, demonstrating the importance of involving primary care physicians in the early detection process. The project’s application to SID, along with its expansion and the development of a federated model for global data sharing, underscores its potential for widespread impact. Collaboration among healthcare providers, researchers, and patient advocacy groups will be key to advancing early detection and improving the management of IEI globally.

In the effort to achieve early detection of IEI, this review highlights the importance of recognizing hematological malignancies as potential indicators of underlying IEI. The hematological manifestations of IEI are multifaceted and require a multidisciplinary approach (hematology, immunology, pathology, and genetics) for effective diagnosis and management. Autoimmune cytopenias, lymphoproliferation, and MDS/BMF each present unique challenges and opportunities for advancing our understanding of IEI. Moreover, genetic and molecular analyses are essential tools in this endeavor, offering the potential for personalized treatment strategies that can significantly improve patient outcomes. As research in this field progresses, it is essential to continue exploring the intricate relationships between IEI and hematological disorders to develop more effective diagnostic and therapeutic approaches.

While this review emphasizes hematologic clues, other non-infectious manifestations —such as allergy, organ-specific autoimmunity, granulomatous-lymphocytic interstitial lung disease, granulomatous dermatitis, arthritis, and inflammatory bowel disease— also warrant attention. Building alliances should therefore extend beyond immunology and hematology to include pulmonology, gastroenterology, rheumatology, dermatology and in a broader way, primary care. A truly multidisciplinary approach is essential to capture the full clinical spectrum of IEI and ensure timely diagnosis and management.

In summary, while significant progress has been made in the early detection of IEI, ongoing efforts to enhance AI applications, improve data quality, and expand successful initiatives remain crucial. In the field of IEI clinical presentation, a multidisciplinary approach involving, among others, primary care physicians, immunologists, hematologists, and pathologists is vital for the early diagnosis and management of underlying immunodeficiencies. This multidisciplinary approach is essential for addressing the complex interplay between IEI, SID, and hematological malignancies.
